# Apologies as signals for change? Implicit theories of personality and reactions to apologies during the #MeToo movement

**DOI:** 10.1371/journal.pone.0226047

**Published:** 2019-12-23

**Authors:** Karina Schumann

**Affiliations:** Department of Psychology, University of Pittsburgh, Pittsburgh, Pennsylvania, United States of America; Valparaiso University, UNITED STATES

## Abstract

During a national reckoning against sexual violence, the public read or heard dozens of apologies offered by prominent public figures in response to allegations of sexual misconduct. This study examined people’s reactions to these apologies, with a focus on whether their implicit theories of personality—their beliefs about whether personality is changeable—influenced their evaluations of the apologies and the men who issued them. Using a nationally representative sample (*N* = 720) and real apologies offered during the #MeToo movement, it was found that, relative to people holding more of an entity (i.e., fixed) view of personality, those holding more of an incremental (i.e., malleable) view evaluated the apologies and apologizers more favorably, held more positive general attitudes toward this recent wave of apologies for misconduct, and were more likely to indicate that redemption was possible for the accused men. These findings suggest that people who hold more of an incremental theory of personality might interpret an apology as a meaningful signal that a person is ready and willing to change their ways and work toward self-improvement.

## Introduction

Amidst the international #MeToo movement against sexual violence that spread virally in 2017, people’s newsfeeds were populated with a continuous stream of sexual misconduct allegations issued against high-profile public figures. Approximately a quarter of these figures offered public apologies in response to the allegations against them, in the hopes that these apologies would help them achieve moral redemption in the eyes of the public. However, the public’s reaction to these apologies likely depended on a variety of factors, including characteristics of the person reacting to the apology (i.e., the target).

One potentially important characteristic is the target’s implicit theory of personality—their belief about whether or not personality can change [1, 2]. People’s beliefs regarding the malleability of personality fall somewhere along a spectrum, ranging from an entity theory at one end to an incremental theory at the other [1]. Whereas people who hold more of an entity theory of personality tend to regard traits as fixed and unchangeable, those who hold more of an incremental theory tend to believe that traits are malleable, and that people have the capacity to change even their most basic qualities.

Past work suggests that these theories have meaningful implications for social judgment. Relative to people who hold more of a fixed view of personality, those who hold more of an incremental view are less likely to believe that past behavior reflects future behavior [1] or make negative trait inferences based on people’s mistakes [3]. These findings suggest that people with more incremental beliefs see past and future behavior as dissociable, and therefore might be particularly receptive to signals that suggest an offender *will* change their behavior. Apologies can act as that signal for change, as they communicate a willingness to take stock of one’s offense and the harm it has caused [4]. Indeed, an apology is theorized to split the offender into two parts: the part that is guilty of the offense and the part that affirms its commitment to better behavior moving forward [5]. People holding a more incremental view might therefore perceive an apology as the start of a quest toward personal change. Consistent with this possibility, past work has demonstrated that intergroup apologies are more effective at producing forgiveness when targets hold a more incremental (vs. more entity) theory of groups [6], that interpersonal apologies are more effective at producing forgiveness for hypothetical offenses when targets hold a more incremental (vs. more entity) theory of personality [7], and that trust recovery is more likely to occur among people receiving an apology if they had previously read an article about morality being malleable [8].

Previous research also suggests that implicit theories of personality predict how people respond to problematic behaviors that call for change. Relative to those holding more of an entity theory, those holding a more incremental theory are more likely to accept responsibility following their own wrongdoings as a way to achieve personal growth [9], and are more likely to confront and constructively voice their dissatisfaction with the wrongdoings of others [10, 11]. This work suggests that people who believe in the malleability of personality prefer actions designed to address and repair offensive behavior, because they believe these actions can result in actual change.

The current study expands on this work by testing whether implicit theories of personality predict reactions to public apologies offered during the #MeToo movement—a context where apologizers had typically been accused of misconduct by multiple accusers, and where doubts about the remorsefulness and morality of the apologizers are high. Although little work has yet been conducted on public apologies for sexual misconduct (for an exception, see [12]), work on other forms of public apologies, such as intergroup and corporate apologies, suggests that these apologies follow a different set of conventions than interpersonal apologies and therefore tend to elicit more skeptical reactions from targets [13]. In particular, because of the public and scripted nature of these apologies [13], as well as doubts about the motives behind them [14], targets are more likely to question the genuineness of these apologies unless they have particular features (e.g., non-verbal expressions of remorse) that communicate sincerity and depth of emotion [13, 15, 16]. Thus, although public apologies are expected and viewed as important [17], they might be viewed as less diagnostic of a transgressor’s sincere willingness to improve their behavior. It is therefore unknown whether people with a more incremental view of personality would still see a public apology offered for sexual misconduct as a meaningful signal for change.

Public apologies—especially those offered by “one-to many” (i.e., when someone in the public eye apologizes publicly for a personal wrongdoing [13], as in the case of #MeToo apologies)—also differ from interpersonal apologies in that they are typically targeted at the broader public in addition to the direct victim(s) of their offense. These apologies are thus intended to help the public figure achieve moral redemption in the eyes of the public, so they might salvage their reputation and ultimately maintain their public support [13]. Although there is almost no work on these types of apologies, a recent set of studies demonstrated that they promote a variety of positive reactions, such as more positive evaluations of, empathy toward, and forgiveness of, the apologizing (vs. non-apologizing) transgressor [13]. These findings suggest that, despite concerns about their sincerity, one-to-many public apologies might still be viewed as important signals for change. If so, the public’s reactions to these types of apologies should be influenced by implicit theories of personality.

The current study tested whether implicit theories of personality are associated with reactions to #MeToo apologies by having a nationally representative sample react to four high-profile apologies that had recently been offered in response to allegations of sexual misconduct. Based on past work showing associations between implicit theories and reactions to apologies in other contexts, and on recent work suggesting that one-to-many apologies are valued by observers, I hypothesized that a more incremental (vs. entity) view of personality would predict more favorable impressions of the apologies (e.g., higher perceived quality and sincerity), the apologizers (e.g., more positive character judgments; greater forgiveness; less punitiveness), and apologies during the #MeToo movement more generally.

## Method

### Participants

A nationally representative sample of 720 participants (366 females, 354 males; *M*_age_ = 46.01, *SD* = 16.69) was recruited from *Qualtrics*, which samples a subset of participants from its pool of over 90 million members. As can be seen in S1 Table, national samples recruited via *Qualtrics* closely approximate U.S. Census estimates of various demographic characteristics and are therefore considered an appropriate method of acquiring nationally representative data [18]. *Qualtrics* also optimizes data quality by building reliability questions (e.g., “For this question, click *strongly* agree”) into the survey; those who fail a reliability question are immediately forwarded to the end of the survey to terminate their participation. The data from those who did not respond reliably were not recorded, however, Qualtrics estimates that fewer than 10% of participants answer unreliably. No additional exclusions were made and all measures collected are reported below. Participants received monetary compensation. These data were collected in December of 2017, when the #MeToo movement was still at its peak and participants were learning of new allegations and apologies on a daily basis. This study was approved by the Institutional Review Board at the University of Pittsburgh (PRO17120360). All participants read an online information letter (a consent form for studies deemed “exempt” due to minimal risk) before participating. All data and materials are available in the data repository on the Open Science Framework at DOI 10.17605/OSF.IO/3U7XS.

A G*Power [19] analysis to detect a medium effect with 90% power required fewer than 100 participants. However, because we were interested in examining the public’s reactions to real world apologies offered during the #MeToo movement, we recruited a larger sample to increase its representativeness. A sensitivity analysis conducted in G*Power showed that this study was powered to detect small effects (Cohen’s *f* = .01).

### Materials and procedure

Participants completed all materials online and were informed that they would be reading a number of real, verbatim statements that had recently been offered by public figures in response to allegations of sexual misconduct. Participants were asked to consider each statement in the context of the allegations that were present at the time the statement was offered, which were listed directly above the statement. In randomized order, participants then read and responded to statements that had been publicly offered by Harvey Weinstein, Kevin Spacey, Russell Simmons, and Al Franken (see S2 Table for full text of each statement; As a comparison, participants also read and responded to a high-profile denial statement offered by Roy Moore. Correlations between implicit theories of personality and reactions to this denial can be found in S3 Table). These statements were selected because they had recently been offered by high profile public figures from different sectors and were diverse in the severity of the allegations against the accused and the content of the statements offered. Although the apologies ranged quite a bit in their comprehensiveness (i.e., the inclusion of apology elements, e.g., acceptance of responsibility; acknowledgment of harm) and degree of defensiveness (i.e., the inclusion of defensive strategies, e.g., justification; minimization) [20], all four apologies included an explicit apology statement (e.g., “I sincerely apologize”). In addition, despite being delivered publicly, all four apologies mentioned the victim(s) in their statement, suggesting that these apologies might be targeted at both the broader public and those who have been directly victimized. All measures below were assessed on 7-point scales (1 = *not at all*, 7 = *to a great degree* or 1 = *strongly disagree*, 7 = *strongly agree*) and are presented in full S1 Appendix.

#### Perceived apology quality

To assess perceived apology quality, participants indicated the extent to which they thought each statement included each of eight apology elements (e.g., “In the above statement, to what extent does Harvey Weinstein express remorse for his actions?”; “In the above statement, to what extent does Harvey Weinstein promise long-term improvements in his behavior?”), and five defensive strategies (e.g., “In the above statement, to what extent does Harvey Weinstein justify his actions?”; “In the above statement, to what extent does Harvey Weinstein deny the alleged behavior?”) [20]. These responses were averaged separately to create indices of *apology comprehensiveness* (Cronbach’s αs ranging from .92-.95; overall *M* = 4.18, *SD* = 1.63; range: *M*_Spacey_ = 3.62, *SD* = 1.52 to *M*_Franken_ = 5.00, *SD* = 1.50) and *defensiveness* (Cronbach’s αs ranging from .82-.90; overall *M* = 3.24, *SD* = 1.49; range: *M*_Franken_ = 2.64, *SD* = 1.50 to *M*_Spacey_ = 3.49, *SD* = 1.37), two features of apology statements that tend to be positively and negatively associated with their effectiveness, respectively [20].

#### Statement evaluations

Participants evaluated each statement with 7 items assessing its sincerity and value (e.g., “This statement seems sincere”; “This statement is insufficient” [reverse-scored]). These items were averaged for each statement to create reliable composites, with Cronbach’s αs ranging from .88-.90 (overall *M* = 3.88, *SD* = 1.50; range: *M*_Weinstein_ = 3.40, *SD* = 1.49 to *M*_Franken_ = 4.59, *SD* = 1.45).

#### Character evaluations

Participants evaluated the accused man’s character with 6 items (e.g., “Harvey Weinstein seems like a good person”; “Harvey Weinstein seems guilty of the allegations against him” [reverse-scored]. These items were averaged for each statement to create reliable composites of character evaluations, with αs ranging from .77-.85 (overall *M* = 3.77, *SD* = 1.25; range: *M*_Weinstein_ = 3.11, *SD* = 1.27 to *M*_Franken_ = 4.23, *SD* = 1.25).

#### Forgiveness

Participants indicated their personal level of forgiveness toward each of the accused men with 5 items (e.g., “I feel forgiving toward Harvey Weinstein”; “I feel anger toward Harvey Weinstein” [reverse-scored]). These items were averaged for each statement to create reliable composites of forgiveness, with αs ranging from .79-.85 (overall *M* = 3.82, *SD* = 1.40; range: *M*_Weinstein_ = 3.20, *SD* = 1.40 to *M*_Franken_ = 4.32, *SD* = 1.41).

#### Punitiveness

Participants responded to 5 items assessing the extent to which they thought the accused should receive various consequences (e.g., “To what extent should Harvey Weinstein be legally punished for his actions?”; “To what extent should Harvey Weinstein be morally redeemed in the eye of the public?” [reverse-scored]). These items were averaged for each statement to create reliable composites of forgiveness, with αs ranging from .77-.85 (overall *M* = 4.56, *SD* = 1.41; range: *M*_Franken_ = 4.11, *SD* = 1.43 to *M*_Weinstein_ = 5.19, *SD* = 1.36).

#### Standardized measure of positive evaluations

The six outcomes described above shared high correlations (see Table 1). Thus, in addition to assessing each outcome separately, we created a standardized measure of all six outcomes (with defensiveness and punitiveness reverse-scored) and tested the association between implicit theories of personality and this standardized measure of overall positive evaluations of each target.

**Table 1:** *Correlations between outcome variables*.

	**Defensiveness**	**Statement Evaluation**	**Character Evaluation**	**Forgiveness**	**Punitiveness**
Comprehensiveness	-.39***	.77***	.56***	.55***	-.50***
Defensiveness	—	-.50***	-.48***	-.40***	.24***
Statement Evaluation		—	.78***	.74***	-.66***
Character Evaluation			—	.82***	-.69***
Forgiveness				—	-.74***
	**Defensiveness**	**Statement Evaluation**	**Character Evaluation**	**Forgiveness**	**Punitiveness**
Comprehensiveness	-.39***	.77***	.56***	.55***	-.50***
Defensiveness	—	-.50***	-.48***	-.40***	.24***
Statement Evaluation		—	.78***	.74***	-.66***
Character Evaluation			—	.82***	-.69***
Forgiveness				—	-.74***
	**Defensiveness**	**Statement Evaluation**	**Character Evaluation**	**Forgiveness**	**Punitiveness**
Comprehensiveness	-.39***	.77***	.56***	.55***	-.50***
Defensiveness	—	-.50***	-.48***	-.40***	.24***
Statement Evaluation		—	.78***	.74***	-.66***
Character Evaluation			—	.82***	-.69***
Forgiveness				—	-.74***

*Note*.

*** *p* < .001.

**General attitudes toward public apologies for misconduct.** After reacting to the five specific statements, participants responded to 5 items assessing their general attitudes toward apologies offered during the #MeToo movement (e.g., “In general, these apologies seem sincere”; α = .76; *M* = 3.26, *SD* = 1.20). Participants also answered 3 items that assessed whether they believed these apologies were beneficial to the apologizer (*M* = 4.73, *SD* = 1.74), to the accusers/victims (*M* = 3.23, *SD* = 1.83), and to raising awareness about the consequences of sexual misconduct (*M* = 4.95, *SD* = 1.72). These items were each analyzed separately.

#### Other means of redemption

As an exploratory measure, participants were asked to “indicate whether there was anything these people can say or do to redeem themselves.” Two independent coders first categorized these responses into the three categories of “yes, redemption might be possible” (*n* = 298, 41%), “no, redemption is not possible” (*n* = 241, 34%) and “other” (which included responses such as being unsure, irrelevant, incoherent, or blank; *n* = 182, 25%). Coders then coded the “yes” responses into 10 categories of how the accused might redeem themselves: self-change (*n* = 140; e.g., “I Believe actions are stronger than words. They need to demonstrate they changed their behavior”), improving the apology (*n* = 123; e.g., “They can own up to their aggressive, immoral behavior and truly apologize for their actions”), making amends directly to victims (*n* = 43; e.g., “Apologize to the accuser in person and listen to them”), advancing the cause (*n* = 37; e.g., “Lead a campaign to fight sexual predation and abuse against women, men and children”), receiving legal punishment (*n* = 32; e.g., “Serve time for what they did”), removing themselves from their position (*n* = 28; e.g., “Remove themselves entirely from positions of power”), payment (*n* = 27; e.g., “Forfeiture of some of their personal wealth payable directly to the victim”), service (*n* = 19; e.g., “Community service, charity work”), the passage of time (*n* = 10; e.g., “Only time will tell”), and religious redemption (*n* = 9; e.g., “Get right with God”). Some responses included more than one way in which the accused might achieve redemption. Reliability between the coders was high (average kappa = .82); discrepancies were resolved by a third coder.

#### Implicit theories of personality and demographics

To assess whether participants believe people can change, we had them complete the 6-item implicit theories of personality scale (e.g., “No matter who somebody is and how they act, they can always change their ways”; α = .87; *M* = 4.25, *SD* = 1.40 [21]. Higher scores on this measure represented a more incremental theory of personality (i.e., the belief that people can change). Finally, participants answered demographic questions and were debriefed via an online feedback letter.

## Results

Because this study used a within-subjects design wherein participants reacted to multiple public apologies, random intercept models were first conducted to calculate intraclass correlations (ICCs) for each of the six dependent variables [22]. Higher ICCs would indicate the presence of stronger correlations between an individual’s ratings of the four different public apologies (e.g., participants who evaluated Al Franken’s apology relatively positively also tended to rate the other apologies relatively positively). These ICC analyses revealed substantial within-subject clustering, with 24%-38% of the variability in the dependent variables being explained by rater (i.e., participant) effects (ICCs: comprehensiveness = 32%; defensiveness = 38%; statement evaluation = 24%; character evaluation = 26%; forgiveness = 37%; punitiveness = 37%). I therefore used multilevel modeling with the four apology statements (level 1) nested within participants (level 2) to test whether participants’ implicit theories of personality predicted their reactions to the apologies on each of the six outcome variables [23].

Linear mixed modeling analyses conducted in SPSS revealed that participants holding more of an incremental (vs. more entity) view of personality indicated more positive reactions to the four apologies in the form of greater perceived apology comprehensiveness, less perceived defensiveness, and more favorable evaluations of the apology (see Table 2 for all test statistics). In addition, participants holding more of an incremental view of personality indicated more positive reactions to the apologizers in the form of more positive evaluations of their character, higher levels of forgiveness, and lower levels of punitiveness. Examining the standardized measure of positive evaluations, we see an overall significant association, with participants holding more of an incremental view of personality reacting more positively to the apology statements and the accused. This association was modest in size, with a pseudo R^2^ of .04 indicating that including implicit theories of personality in the model resulted in a 4% reduction in unexplained variance (compared to an intercept-only model). Correlations between implicit theories of personality and the outcomes for each separate statement are presented in S3 Table in Supplementary Materials.

**Table 2:** Test statistics for associations with implicit theories of personality.

	*Estimate (SE)*	*df*	*T value*	*P value*	*95% CI*	*Pseudo R*^*2*^
Comprehensiveness	.13 (.030)	717.96	4.47	< .001	.0754, .1935	.01
Defensiveness	-.14 (.029)	717.19	-4.99	< .001	-.1989, -.0866	.02
Statement Evaluation	.13 (.026)	717.94	4.81	< .001	.0738, .1758	.01
Character Evaluation	.16 (.022)	718.10	7.49	< .001	.1188, .2033	.03
Forgiveness	.20 (.026)	718.14	7.65	< .001	.1487, .2515	.04
Punitiveness	-.14 (.027)	718.62	-5.14	< .001	-.1903, -.0850	.02
**Stzd Positive Evaluations**	**.11 (.015)**	**717.99**	**7.26**	**< .001**	**.0802, .1397**	**.04**

*Note*. Parameter estimates are unstandardized.

Examining participants’ general attitudes toward the recent wave of apologies for sexual misconduct, those holding a more incremental view of personality reported more positive attitudes toward these apologies (*r* = .23, *p* < .001). Although small in magnitude, those with more incremental views also rated the apologies as being more beneficial to the accuser/victims (*r* = .09, *p* = .016) and to raising awareness about the consequences of sexual misconduct (*r* = .11, *p* = .003), but not as more beneficial to the apologizers (*r* = .02, *p* = .644).

Finally, implicit theories of personality were related to participants’ responses to the open-ended question asking whether the accused could do anything to redeem himself. Binary logistic regressions were first used to assess whether implicit theories of personality predicted responses that were coded as indicating “no, change is not possible” (coded as 0) versus “yes, change might be possible” (coded as 1). This analysis revealed that those with more incremental views were significantly more likely to indicate that redemption was possible, *B* = .37, *SE* = .07, Wald *χ*^*2*^(1) = 31.66, OR = 1.45, 95% CI = 1.27–1.65, *p* < .001. Looking only within the “yes” category at the specific ways in which the accused might redeem themselves, those with more incremental views were more likely to indicate self-change (*B* = .18, *SE* = .09, Wald *χ*^*2*^(1) = 4.35, OR = 1.19, 95% CI = 1.01–1.41, *p* = .037) as a means to redemption. This finding is consistent with the incremental belief that change is possible. No other ways of achieving redemption were associated with implicit theories, all *p*s > .154.

## Discussion

Public apologies have become a daily occurrence, with the #MeToo movement representing a particularly important context in which the world witnessed a deluge of apologies offered by prominent figures. Although it remains to be seen whether any of the accused will be morally redeemed in the eye of the public, the current study sheds some light on features of the public that influence how these apologies have been received. Across multiple real #MeToo apologies and various outcomes, the results painted a consistent picture: people with a more incremental (vs. more entity) view of personality showed more positive reactions to these apologies and the people offering them. Although these associations were somewhat small in magnitude, they remain meaningful in a context where apologies are generally perceived as insincere due to their prevalence [17], their public nature [13], the severity of the offenses [24], and the number of accusations against each of the accused. These contextual factors likely limit the extent to which apologies are interpreted as sincere signals of future change. Consistent with this skepticism, on average, participants tended to hold somewhat negative views of the apologies and apologizers, falling below the midpoint on statement evaluations, character evaluations, forgiveness, and general attitudes toward #MeToo apologies, while falling above the midpoint on punitiveness.

However, the current findings suggest that—despite these contextual factors—people holding a more incremental view of personality are more likely to interpret an apology as a meaningful cue that the accused individual is ready and willing to initiate a process of self-change. By contrast, those holding a more entity view of personality might be more likely see an apology as empty words—words that will exert no meaningful impact on future behavior because change is not possible. This work therefore extends past work by revealing the influence of implicit theories in a real-world context where there is high skepticism about an offender’s ability or willingness to change. With the view that change is possible, an apology takes on a different meaning. Further, it extends work on public apologies by being one of only several papers examining the increasingly prevalent one-to-many type of public apology [13], and by identifying a factor that influences the effectiveness of public apologies. This is important because past work has produced mixed results regarding the effectiveness of apologies that are delivered publicly (e.g., corporate apologies; apologies by government officials) [15, 16]. The current study suggests that, despite skepticism about the genuineness of these public apologies, they might function similarly to interpersonal apologies and be influenced by similar individual difference predictors.

The current study demonstrated that implicit theories of personality predicted a range of outcomes, including evaluations of the apology, forgiveness, and punitiveness toward the accused men. Although much of the philosophical literature on forgiveness assumes that only those directly hurt by the transgression have the right to forgive the transgressor [16], this view has been recently challenged by those who believe that people not directly harmed can also experience hostile reactions to an offense, reactions that can then be forgiven [25]. Empirically, recent work has demonstrated that although victims (in the form of members of the victimized group) are less likely than non-victims to forgive transgressors, both victims and non-victims are more forgiving following a public apology compared to no apology [13]. These findings suggest that, at least in the context of apologies that are offered to the broader public, non-victims might feel they have a right to forgive because (a) they perceive that at least part of the apology is being directed at them [13], (b) they feel morally outraged by the transgression [25], or (c) they feel directly victimized because they believe the public figure has violated an obligation to the public to behave morally due to their visibility and influence. More work is needed to understand the psychology of being a member of the public receiving a one-to-many public apology, including whether and why they believe they have a right to forgive the apologizer.

One of the major strengths of this study is that it aimed to approximate the conditions under which people evaluate real public statements. Participants read multiple statements during one session and knew who had issued these statements. These statements also varied considerably in content and had been issued in response to accusations that varied in severity and other important dimensions. This method allowed us to capture people’s reactions in a highly realistic manner, but carried the limitation of reducing our control over these various dimensions. These four statements were also selected from dozens of #MeToo apologies that had been delivered at the time this study was conducted. Although they were selected to capture a range of features of the apology, apologizer, and context (e.g., severity of the allegations), they may not be representative of apologies offered during the #MeToo movement.

Future work might examine how implicit theories of personality influence reactions to public apologies that are substantiated by actual efforts to change one’s behavior, such as seeking out therapy or becoming involved in causes that allow the accused to learn about victims’ experiences. Future work might also examine whether apologizers across various contexts might bolster reactions to their apologies by directly addressing their capacity to change and their specific plans for how they will do so, as well as whether addressing their plans for self-change garners different reactions from people with more incremental vs. more fixed theories of personality. Finally, future work might examine whether there are contextual factors that moderate incremental theorists’ optimism for change following an apology. Is there a point at which apologies become meaningless even to people with more incremental views? As noted above, the context of #MeToo presents many factors (e.g., whether the accused has multiple allegations against him; the severity of those allegations) that likely mitigate the effectiveness of any apologies offered. However, a systematic examination of how these types of factors interact with implicit theories of personality to affect reactions would provide greater insight into when and for whom apologies are effective.

## Supporting information

S1 Appendix Complete study materials. (PDF)

S1 Table Demographic comparison of 2015 U.S. census bureau data and Qualtrics sample. (PDF)

S2 Table Full text of public statements used in current study. (PDF)

S3 Table Correlations between implicit theories of personality and the outcomes for each statement. (PDF)
