# Supplementary material for: Apologies as signals for change? Implicit theories of personality and reactions to apologies during the #MeToo movement
Source: PLoS One. 2019 Dec 23;14(12):e0226047. doi: 10.1371/journal.pone.0226047 (PMC6927633; doi:10.1371/journal.pone.0226047)
Supplement: S1 Appendix — (PDF) [file pone.0226047.s001.pdf]

S1 Appendix

Complete Study Measures

Public Apologies for Sexual Misconduct Study

In this study, you will read a number of real, verbatim statements that have recently been offered by public figures in response to allegations of sexual misconduct (ranging from sexual harassment to rape). Although some of these public figures have been accused of other acts since the time of these statements, please consider each statement in the context of the allegations that were present at the time the statement was offered (this information is provided above each statement).

[Demographic questions for selection of nationally representative sample]

1. Gender: _____ Male _____ Female _____ Other (please specify): ___________________

2. Age: ____________________

3. Please specify your age category:

- ◯ Under 18
- ◯ 18-24
- ◯ 25 - 34
- ◯ 35 - 44
- ◯ 45 - 54
- ◯ 55 - 64
- ◯ 65+

4. Ethnicity:

- ◯ White
- ◯ Black or African American
- ◯ American Indian or Alaskan Native
- ◯ Asian
- ◯ Hispanic
- ◯ Other

5. Please specify your income:

- ◯ $0- < $25K
- ◯ $25K- < $50K
- ◯ $50K- < $75K
- ◯ $75K- < $100K
- ◯ $100K- < $150K
- ◯ $150K- < $200K
- ◯ $200K+

[In randomized order, participants saw the five statements presented in S2 Table. After reading each statement they answered the following questions]

Please use the scale below to answer the questions that follow:

1	2	3	4	5	6	7
Not at all			Somewhat			To a great degree

In the above statement, to what extent does [accused]:

1. Express remorse for his actions?
2. Accept responsibility for his actions?
3. Offer some form of repair for his actions?
4. Promise long-term improvements in his behavior?
5. Ask for forgiveness?
6. Admit that his actions were wrong or immoral?
7. Acknowledge causing harm to others?
8. Explain his actions *without* pushing blame away from himself?
9. Deny the alleged behavior?
10. Blame the person(s) accusing him?
11. Shift blame away from himself?
12. Justify his actions?
13. Minimize the severity of his actions?

[Items 1-8: apology elements used in measure of *Apology Comprehensiveness*; Items 9-13: defensive strategies used in measure of *Defensiveness*]

Please describe your reaction to this person's statement using the scale provided:

1	2	3	4	5	6	7
Strongly Disagree			Neither agree nor disagree			Strongly Agree

1. This statement seems sincere.
2. This statement is insufficient. [R]
3. This statement is satisfying.
4. This statement is worthless. [R]
5. This statement improves my perceptions of [accused].
6. This statement worsens my perceptions of [accused]. [R]
7. I believe what [accused] says in his statement.
8. I believe that [accused] will commit similar acts in the future. [R]
9. Nothing [accused] could say would be able to change my opinion of him. [R]
10. [Accused] seems guilty of the allegations against him. [R]
11. [Accused] seems like a good person.
12. [Accused] seems like an immoral person. [R]
13. [Accused] seems like he could change for the better.
14. [Accused] disgusts me. [R]
15. I feel compassion for [accused].
16. I feel anger toward [accused]. [R]
17. I feel forgiving toward [accused].
18. The allegations against [accused] are unforgivable. [R]

[Items 1-7: Items used in measure of *Statement Evaluations*; Items 8-13: Items used in measure of *Character Evaluations*; Items 14-18: Items used in measure of *Forgiveness*]

Based on the above statement, to what extent:

1	2	3	4	5	6	7
Not at all			Somewhat			To a great degree

1. Should [accused] be forgiven by his accusers for his actions? [R]
2. Should [accused] be legally punished for his actions?
3. Should [accused] be morally redeemed in the eye of the public? [R]
4. Should [accused] be removed from the position he held at the time of these allegations?
5. Should [accused] be allowed to work in a similar position in the future? [R]

[Items 1-5: Items used in measure of *Punitiveness*]

[After participants offered reactions to all five statements, they answered the following questions about public apologies for sexual misconduct more generally]

The questions below ask you about your general thoughts regarding the apologies that have recently been offered by public figures for sexual misconduct.

1	2	3	4	5	6	7
Strongly Disagree			Neither agree nor disagree			Strongly Agree

1. In general, these public apologies seem sincere.
2. In general, these public apologies seem manipulative. [R]
3. In general, there is nothing that these people can say to redeem themselves. [R]
4. In general, it seems like these people are just apologizing because they have to. [R]
5. The large number of apologies being offered makes each apology less meaningful. [R]
6. In general, these public apologies seem beneficial for the apologizers.
7. In general, these public apologies seem beneficial for the accusers/victims.
8. In general, these public apologies seem beneficial to raising awareness about the consequences of sexual misconduct.

[Items 1-5: Items used in measure of *General attitudes toward public apologies for misconduct*; Items 6-8 each analyzed separately]

Please indicate whether there is anything you think these people can say or do to redeem themselves:

____

____

____

____

[Responses coded for *Other Means of Redemption*]

We are now going to ask you some questions about you.

Using the scale below, please indicate your agreement with each of the following statements. There are no right or wrong answers.

1	2	3	4	5	6	7
Strongly Disagree						Strongly Agree

1. People can't really change what kind of personality they have. Some people have a good personality and some don't and they can't change much.
2. Someone's personality is a part of them that they can't change very much.
3. A person can do things to get people to like them, but they can't change their real personality.
4. No matter who somebody is and how they act, they can always change their ways.
5. Anybody can change their personality a lot.
6. People can always change their personality.

Please provide the following information in order to help us identify any background factors that may be related to our findings. ***All*** *information provided will be kept confidential.*

1. Country of Birth: ______________________

2. First language: ________________________

3. How would you describe your political identity?

____ Democrat ____ Republican ____ Independent _____ Other (please specify):

4. What is your political orientation?

1	2	3	4	5	6	7
Extremely liberal						Extremely conservative

8. Religion (if any): ________________________

9. How religious are you?

1	2	3	4	5	6	7
Not at all religious						Extremely religious
