# Supplementary material for: Apologies as signals for change? Implicit theories of personality and reactions to apologies during the #MeToo movement
Source: PLoS One. 2019 Dec 23;14(12):e0226047. doi: 10.1371/journal.pone.0226047 (PMC6927633; doi:10.1371/journal.pone.0226047)
Supplement: S1 Table — (PDF) [file pone.0226047.s002.pdf]

Comparison of study demographics to population demographics

To demonstrate the representativeness of the *Qualtrics* sample used in the current study, S1 Table compares the distributions of gender, age, ethnicity, and income of the current sample to population distributions obtained from the 2015 U.S. Census. All categories were within an acceptable range of less than 5% discrepancy (M = .88%), suggesting that the demographic breakdown for this sample closely approximated the demographic breakdown of the national population.

S1 Table
Demographic comparison of 2015 U.S. census bureau data and Qualtrics sample

Demographic Variable	2015 U.S. Census Data	Qualtrics Sample
**Gender** (18+)		
Female	51.3%	50.8%
Male	48.7%	49.2%
**Age** (years)		
18 - 24	12.6%	12.8%
25 - 34	17.8%	17.6%
35 - 44	16.4%	16.7%
45 - 54	17.4%	17.8%
55 - 64	16.5%	16.4%
65 and over	19.3%	18.8%
**Race/Ethnicity** (18+)		
White	64.6%	61.9%
Black	12.0%	12.4%
Hispanic	15.5%	17.4%
Asian	5.6%	5.3%
American Ind./AK Native	0.7%	0.7%
Other	1.6%	2.4%
**Household Income**		
$0 to < $25,000	22.1%	17.6%
$25,000 to < $50,000	22.7%	22.5%
$50,000 to < $75,000	16.7%	19.0%
$75,000 to < $100,000	12.1%	13.6%
$100,000 to < $150,000	14.1%	15.1%
$150,000 to < $200,000	6.2%	6.0%
$200,000 and above	6.1%	6.1%

Note. Population estimates for gender, age, and race/ethnicity derived from the U.S. Census Bureau, Population Division: April 1, 2010 to July 1, 2015. Retrieved from https://factfinder.census.gov/faces/tableservices/jsf/pages/productview.xhtml?src=bkmk.
Population estimate for household income derived from the U.S. Census Bureau, Current Population Survey: 2015 Annual Social and Economic Supplement. Retrieved from https://www.census.gov/data/tables/time-series/demo/income-poverty/cps-hinc/hinc-01.2015.html
