# Supplementary material for: Apologies as signals for change? Implicit theories of personality and reactions to apologies during the #MeToo movement
Source: PLoS One. 2019 Dec 23;14(12):e0226047. doi: 10.1371/journal.pone.0226047 (PMC6927633; doi:10.1371/journal.pone.0226047)
Supplement: S2 Table — (PDF) [file pone.0226047.s003.pdf]

S2 Table

Full text of public statements used in current study

HARVEY WEINSTEIN

Source: USA Today

Retrieved from: https://www.usatoday.com/story/life/movies/2017/10/05/harvey-weinstein-scandal-read-his-full-apology/738093001/

Harvey Weinstein, Producer and co-founder of large Hollywood company
Allegations at time of statement: Accused by least 8 people of sexual harassment and coercion, inappropriate touching
Statement: "I came of age in the '60s and '70s, when all the rules about behavior and workplaces were different. That was the culture then. I have since learned it's not an excuse, in the office — or out of it. To anyone. I realized some time ago that I needed to be a better person, and my interactions with the people I work with have changed. I appreciate the way I've behaved with colleagues in the past has caused a lot of pain, and I sincerely apologize for it. Though I'm trying to do better, I know I have a long way to go. That is my commitment. My journey now will be to learn about myself and conquer my demons. Over the last year, I've asked Lisa Bloom to tutor me, and she's put together a team of people. I've brought on therapists, and I plan to take a leave of absence from my company and to deal with this issue head on. I so respect all women, and regret what happened. I hope that my actions will speak louder than words and that one day we will all be able to earn their trust and sit down together with Lisa to learn more. Jay Z wrote in 4:44 'I'm not the man I thought I was, and I better be that man for my children.' The same is true for me. I want a second chance in the community, but I know I've got work to do to earn it. I have goals that are now priorities. Trust me, this isn't an overnight process. I've been trying to do this for 10 years, and this is a wake-up call. I cannot be more remorseful about the people I hurt, and I plan to do right by all of them. I am going to need a place to channel that anger, so I've decided that I'm going to give the NRA my full attention. I hope Wayne LaPierre will enjoy his retirement party. I'm going to do it at the same I had my Bar Mitzvah. I'm making a movie about our President, perhaps we can make it a joint retirement party. One year ago, I began organizing a $5 million foundation to give scholarships to women directors at USC. While this might seem coincidental, it has been in the works for a year. It will be named after my mom, and I won't disappoint her."

KEVIN SPACEY

Source: CNN

Retrieved from: https://www.cnn.com/2017/10/30/entertainment/kevin-spacey-allegations-anthony-rapp/index.html

Kevin Spacey, Actor
Allegations at time of statement: Accused by one person of sexual assault of a minor
Statement: "I have a lot of respect and admiration for Anthony Rapp as an actor. I'm beyond horrified to hear his story. I honestly do not remember the encounter, it would have been over 30 years ago. But if I did behave then as he describes, I owe him the sincerest apology for what would have been deeply inappropriate drunken behavior, and I am sorry for the feelings he describes having carried with him all these years. This story has encouraged me to address other things about my life. I know that there are stories out there about me and that some have been fueled by the fact that I have been so protective of my privacy. As those closest to me know, in my life I have had relationships with both men and women. I have loved and had romantic encounters with men throughout my life, and I choose now to live as a gay man. I want to deal with this honestly and openly and that starts with examining my own behavior."

RUSSELL SIMMONS

Source: Deadline

Retrieved from: https://deadline.com/2017/11/russell-simmons-exit-sexual-harassment-jenny-lumet-1202217311/

Russell Simmons, Entrepreneur, (music) producer, author
Allegations at time of statement: Accused by one person of sexual assault, rape
Statement: "I have been informed with great anguish of Jenny Lumet's recollection about our night together in 1991. I know Jenny and her family and have seen her several times over the years since the evening she described. While her memory of that evening is very different from mine, it is now clear to me that her feelings of fear and intimidation are real. While I have never been violent, I have been thoughtless and insensitive in some of my relationships over

many decades and I sincerely and humbly apologize. This is a time of great transition. The voices of the voiceless, those who have been hurt or shamed, deserve and need to be heard. As the corridors of power inevitably make way for a new generation, I don't want to be a distraction so I am removing myself from the businesses that I founded. The companies will now be run by a new and diverse generation of extraordinary executives who are moving the culture and consciousness forward. I will convert the studio for yogic science into a not-for-profit center of learning and healing. As for me, I will step aside and commit myself to continuing my personal growth, spiritual learning and above all to listening."

AL FRANKEN

Source: CNN

Retrieved from: http://www.cnn.com/2017/11/16/politics/al-franken-apology/index.html

Al Franken, Senator (Dem) for Minnesota
Allegations at time of statement: Accused by 1 person of sexual misconduct (inappropriate touching)
Statement: "The first thing I want to do is apologize: to Leeann, to everyone else who was part of that tour, to everyone who has worked for me, to everyone I represent, and to everyone who counts on me to be an ally and supporter and champion of women. There's more I want to say, but the first and most important thing—and if it's the only thing you care to hear, that's fine—is: I'm sorry. I respect women. I don't respect men who don't. And the fact that my own actions have given people a good reason to doubt that makes me feel ashamed. But I want to say something else, too. Over the last few months, all of us—including and especially men who respect women—have been forced to take a good, hard look at our own actions and think (perhaps, shamefully, for the first time) about how those actions have affected women. For instance, that picture. I don't know what was in my head when I took that picture, and it doesn't matter. There's no excuse. I look at it now and I feel disgusted with myself. It isn't funny. It's completely inappropriate. It's obvious how Leeann would feel violated by that picture. And, what's more, I can see how millions of other women would feel violated by it—women who have had similar experiences in their own lives, women who fear having those experiences, women who look up to me, women who have counted on me. Coming from the world of comedy, I've told and written a lot of jokes that I once thought were funny but later came to realize were just plain offensive. But the intentions behind my actions aren't the point at all. It's the impact these jokes had on others that matters. And I'm sorry it's taken me so long to come to terms with that. While I don't remember the rehearsal for the skit as Leeann does, I understand why we need to listen to and believe women's experiences. I am asking that an ethics investigation be undertaken, and I will gladly cooperate. And the truth is, what people think of me in light of this is far less important than what people think of women who continue to come forward to tell their stories. They deserve to be heard, and believed. And they deserve to know that I am their ally and supporter. I have let them down and am committed to making it up to them."

ROY MOORE

Source: WJLA Washington

Retrieved from: http://wjla.com/news/nation-world/watch-live-roy-moore-makes-statement-following-sexual-misconduct-allegations

Roy Moore, Retired Chief Justice of Alabama Supreme Court, candidate for US Senate
Allegations at time of statement: Accused by one person of sexual touching of a minor; Accused by three additional people of pursuing them sexually as minors
Statement: "I want to address what's been brought out on television today by Gloria Allred and the client that she has about things I allegedly did. I want to make it perfectly clear. The people of Alabama know me, they know my character, they know what I've stood for in the political world for over 40 years. I can tell you without hesitation this is absolutely false. I never did what she said I did. I don't even know the woman. I don't know anything about her. I don't even know where the restaurant is or was. This is a political maneuver. It has nothing to do with reality."

Note. Information regarding the accused individual's position and allegations at the time of the statement were provided to participants above each statement. Statements were presented in randomized order.
