# Supplementary material for: Apologies as signals for change? Implicit theories of personality and reactions to apologies during the #MeToo movement
Source: PLoS One. 2019 Dec 23;14(12):e0226047. doi: 10.1371/journal.pone.0226047 (PMC6927633; doi:10.1371/journal.pone.0226047)
Supplement: S3 Table — (PDF) [file pone.0226047.s004.pdf]

Correlations between implicit theories of personality and the outcomes for each statement

In addition to the four apologies presented in the manuscript, participants also read and reacted to a denial by Roy Moore. Participants rated this denial statement as relatively low in comprehensiveness (M = 1.79, SD = 1.46) and high in defensiveness (M = 4.84, SD = 1.46). Participants also expressed relatively unfavorable attitudes toward Moore's statement (M = 2.71, SD = 1.58) and Moore ($M_{\text{character\_evaluations}}$ = 3.08, SD = 1.45; ($M_{\text{forgiveness}}$ = 3.03, SD = 1.54; $M_{\text{punitiveness}}$ = 5.11, SD = 1.63).

S3 Table presents associations with implicit theories of personality for each of the four apology statements and Moore's denial separately. These correlations do not account for clustering at the participant level, but reveal how implicit theories of personality relate to reactions to each of the four apologies and the denial separately. Implicit theories of personality were largely correlated with more positive evaluations of each of the apologies and apologizers, but largely uncorrelated with reactions to Roy Moore's denial statement. However, several notable exceptions emerged. First, implicit theories were uncorrelated with several indices of reactions to Spacey's apology, possibly because his apology included a denial statement in it (see S2 Table). Second, implicit theories were positively correlated with judgments of Moore's character and forgiveness toward him, although these correlations were weaker than those observed for the four apologizers. It therefore appears that—because they are less likely to make trait inferences based on negative behavior—those who hold a more incremental view of personality might generally see people more positively after their offenses, even in the absence of an apology. However, when examining the standardized measure of positive evaluations, we see the expected significant correlations between implicit theories of personality and the four apologies, and no correlation between implicit theories of personality and the denial.

S3 Table

Correlations between implicit theories of personality and outcomes for each statement

	Al Franken	Harvey Weinstein	Kevin Spacey	Russell Simmons	Roy Moore
Comprehensiveness	$.14^{***}$	$.17^{***}$	.03	$.13^{***}$	-.07
Defensiveness	$-.16^{***}$	$-.17^{***}$	$-.10^{**}$	$-.12^{**}$	-.01
Statement Evaluation	$.13^{**}$	$.16^{***}$	.04	$.16^{***}$	.03
Character Evaluation	$.16^{***}$	$.19^{***}$	$.20^{***}$	$.22^{***}$	$.11^{**}$
Forgiveness	$.19^{***}$	$.23^{***}$	$.20^{***}$	$.21^{***}$	$.10^{**}$
Punitiveness	$-.14^{***}$	$-.16^{***}$	$-.13^{**}$	$-.15^{***}$	-.06
**Stzd Positive Evaluations**	**$.19^{***}$**	**$.23^{***}$**	**$.15^{***}$**	**$.21^{***}$**	**.05**

Note. *** $p < .001$; ** $p < .01$.
